# The Tri-Trophic Interactions Hypothesis: Interactive Effects of Host Plant Quality, Diet Breadth and Natural Enemies on Herbivores

**DOI:** 10.1371/journal.pone.0034403

**Published:** 2012-04-11

**Authors:** Kailen A. Mooney, Riley T. Pratt, Michael S. Singer

**Affiliations:** 1 Department of Ecology and Evolutionary Biology, University of California Irvine, Irvine, California, United States of America; 2 Department of Biology, Wesleyan University, Middletown, Connecticut, United States of America; Umea University, Sweden

## Abstract

Several influential hypotheses in plant-herbivore and herbivore-predator interactions consider the interactive effects of plant quality, herbivore diet breadth, and predation on herbivore performance. Yet individually and collectively, these hypotheses fail to address the simultaneous influence of all three factors. Here we review existing hypotheses, and propose the tri-trophic interactions (TTI) hypothesis to consolidate and integrate their predictions. The TTI hypothesis predicts that dietary specialist herbivores (as compared to generalists) should escape predators and be competitively dominant due to faster growth rates, and that such differences should be greater on low quality (as compared to high quality) host plants. To provide a preliminary test of these predictions, we conducted an empirical study comparing the effects of plant (*Baccharis salicifolia*) quality and predators between a specialist (*Uroleucon macolai*) and a generalist (*Aphis gossypii*) aphid herbivore. Consistent with predictions, these three factors interactively determine herbivore performance in ways not addressed by existing hypotheses. Compared to the specialist, the generalist was less fecund, competitively inferior, and more sensitive to low plant quality. Correspondingly, predator effects were contingent upon plant quality only for the generalist. Contrary to predictions, predator effects were weaker for the generalist and on low-quality plants, likely due to density-dependent benefits provided to the generalist by mutualist ants. Because the TTI hypothesis predicts the superior performance of specialists, mutualist ants may be critical to *A. gossypii* persistence under competition from *U. macolai*. In summary, the integrative nature of the TTI hypothesis offers novel insight into the determinants of plant-herbivore and herbivore-predator interactions and the coexistence of specialist and generalist herbivores.

## Introduction

For more than half a century, evolutionary ecologists have studied plant-herbivore interactions with the dual aims of understanding plant defense and dietary specialization by insect herbivores [1], [2], [3], [4], [5], [6], [7], [8]. While most research before 1980 viewed plant-herbivore interactions from a bi-trophic perspective – considering plant defense and herbivore offense alone – the role of predators and parasitoids (natural enemies) has increasingly been recognized as important [9], [10], [11], [12], [13]. For plants, natural enemies can serve as indirect plant defenses and can mediate the efficacy of direct defenses [11], [14], [15]. For herbivores, natural enemies play a central role in shaping the trade-offs between the costs and benefits of broad vs. narrow diet breadths [12], [16].

**Figure 1:**
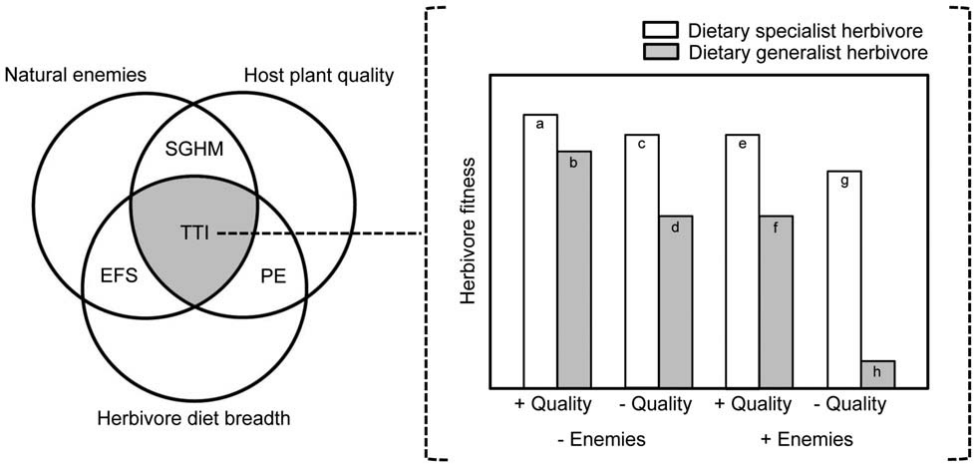
Predictions of the tri-trophic interactions (TTI) hypothesis for the interactive effects of natural enemies, host-plant quality and diet breadth on herbivores. Three well-studied hypotheses – the physiological efficiency (PE), enemy free space (EFS) hypotheses, and slow-growth/high-mortality (SGHM) – each address unique, pairwise combinations of these factors. The physiological efficiency (PE) hypothesis predicts specialists should outperform generalists on shared host plants (e.g. a>b), and that generalists should be more sensitive to variation in host-plant quality than specialists (e.g. a–c<b–d). The Enemy Free Space (EFS) hypothesis predicts natural enemies should have a stronger effect on dietary specialists than generalists (e.g. a–e<b–f). The Slow-Growth/High-Mortality (SGHM) hypothesis predicts low host-plant quality enhances the effects of natural enemies (e.g. b–f<d–h). The TTI hypothesis offers novel predictions for the three-way interaction among these factors: Dietary specialists (as compared to generalists) are predicted to escape natural enemies and be competitively dominant due to faster growth rates, and such differences should be greater on low quality (as compared to high quality) host plants. Such non-additive dynamics imply that predictions for the PE, EFS, and SGHM hypotheses are contingent upon the third, discounted factor. Natural enemies should mediate the predictions of the PE hypothesis, such that the differential effects of host-plant quality on specialists and generalists is greater in the presence of natural enemies (e–g≪f–h) than in the absence of natural enemies (a–c<b–d). Host-plant quality should mediate the predictions of the EFS hypothesis, such that the differential effects of natural enemies on specialist and generalist herbivores is greater on low-quality host plants (c–g≪d–h) than on high-quality host plants (a–e<b–f). Herbivore diet breadth should mediate the predictions of the SGHM hypothesis, such that SGHM dynamics are stronger for dietary generalist (b–d≪f–h) than specialist herbivores (a–c<e–g).

While a multi-trophic approach has been applied to many aspects of plant-herbivore interactions (reviewed by [17], [18], [19]), here we address the dual influences of natural enemies and host plant quality on the relative performance and coexistence of dietary specialist and generalist herbivores. In the large literature on this topic, three long-standing hypotheses are especially relevant: The physiological efficiency [1], slow-growth/high-mortality [11], [20], [21] and enemy-free space hypotheses [22], [23]. These highly influential hypotheses are notable for their integrative nature, with each addressing the interactive effects of unique pairwise combinations of host-plant quality, natural enemies and herbivore diet breadth upon herbivore performance (Fig. 1, Table 1).

**Table 1:** Descriptions of three long-standing hypotheses for plant-herbivore and herbivore-predator interactions and their relation to the tri-trophic interactions hypothesis.

Original hypothesis			Predictions under tri-trophic interactions hypothesis
Name	Factors considered	Predictions	
Physiological efficiency	Diet breadth, plant quality	Specialists are better adapted than generalists at using shared plants as food (a>b, c>d, e>f, and g>h) and variation in host-plant quality should have stronger effects on generalists than specialists (a–c<b–d and e–g<f–h)	The benefits of specialization for performance are greater in the presence of natural enemies (e–g≪f–h) than absence of natural enemies (a–c<b–d)
Enemy-free space	Diet breadth, natural enemies	Specialist are better than generalists at using shared plants for predator avoidance (a–e<b–f and c–g<d–h)	The benefits of specialization for predator avoidance are greater on low-quality plants (c–g≪d–h) than high-quality plants (a–e<b–f)
Slow-growth/high-mortality	Plant quality, natural enemies	Low plant quality increases the effects of natural enemies (a–e<c–g and b–f<d–h)	Low plant quality increases the effects of natural enemies more for generalists (b–f≪d–h) than specialists (a–e<c–g)

Parenthetical references to a–h refer to the graphical representation of these predictions shown in Fig. 1.

In this paper, we extend this multi-trophic approach to consider the simultaneous effects of all three factors upon both herbivore performance and coexistence. We first provide a brief overview of these three long-standing hypotheses and their predictions, with the primary goal of illustrating areas of overlap and differentiation. While the appeal of these hypotheses is their integrative nature, they are also splintered, and collectively fail to consider the simultaneous and interactive effects among host-plant quality, herbivore diet breadth and natural enemies. Accordingly, we propose and describe the tri-trophic interactions hypothesis, consolidating the predictions of existing hypotheses. We then empirically test this new hypothesis, and conclude by discussing future directions for its further development and testing.

### Current hypotheses

The physiological efficiency (PE) hypothesis states that dietary specialists are better adapted than generalists at physiologically utilizing their host plants as food [1]. As a result, specialists should have superior physiological performance (e.g., more efficient resource assimilation and faster growth rates) than generalists on their shared host plants [24], [25]. As depicted in Fig. 1, the PE hypothesis predicts a>b, c>d, e>f, and g>h. This central prediction of the PE hypothesis has found support in some (e.g. [26]) but not all (e.g. [24]) studies (reviewed by [27]).

The PE hypothesis also offers three predictions for the interactive effects of host-plant quality and herbivore diet breadth. First, variation in host-plant quality should have stronger effects on dietary generalists than on better-adapted dietary specialists [28]. As depicted in Fig. 1, the PE hypothesis predicts a–c<b–d (PE effects without natural enemies) and e–g<f–h (PE effects with natural enemies). However, the PE hypothesis does not offer predictions for the relative magnitude of PE effects between the presence and absence of natural enemies. Past studies support this first prediction, showing that toxic forms of plant secondary compounds have larger effects on the performance of generalist than specialist herbivores, while generalist and specialist herbivores performed more similarly on less toxic plants [26], [28]. Second, differences in performance associated with diet breadth in turn imply asymmetrical competition, such that specialists should competitively dominate generalists [29], [30] (not depicted in Fig. 1). Competition appears to be especially important in structuring communities of sap- and internally-feeding herbivores [31], [32]. Therefore, a third PE prediction for such herbivores is that competitive asymmetries between generalists and specialists should be more pronounced on low- than high-quality host plants (not depicted in Fig. 1). While some studies have shown host plant quality to mediate herbivore competition (e.g. [33], [34], [35], reviewed by [32]), others have not (e.g. [36], [37]), and most tests for variation in competition among plant genotypes show no such effect (reviewed by [38]). No studies, to our knowledge, have tested the effects of herbivore diet breadth on competitive asymmetries.

The enemy-free space hypothesis (EFS) considers the interaction between herbivore diet breadth and natural enemies on herbivore performance. It states that specialist herbivores are better adapted than generalists at using their host plants for protection or defense from predators due to their superior crypsis (chemical or visual) or ability to sequester plant secondary compounds for their own defense [12]. The main prediction of this hypothesis is reduced predation rates of specialists as compared to generalists on their shared host plants. As depicted in Fig. 1, the EFS hypothesis predicts a–e<b–f (EFS effects on high-quality plants) and c–g<d–h (EFS effects on low-quality plants). However, the EFS hypothesis does not offer predictions for the relative magnitude of EFS effects between low- and high-quality plants. Previous studies have largely supported the main prediction of EFS advantages to specialists (e.g. [39], [40], [41]), but generalist herbivores may also use host plants (e.g. [42]) or mutualists (e.g. [43]) in defense against natural enemies in ways that confound the main prediction of the EFS hypothesis.

Finally, the slow-growth/high-mortality hypothesis (SGHM) considers the interaction between host-plant quality and natural enemies. It intuitively proposes that herbivore development on a poor quality host plant will be relatively slow, extending the duration of juvenile phases that are the most vulnerable to natural enemies [4], [11], [20], [44]. As depicted in Fig. 1, the SHGM hypothesis predicts a–e<c–g (SGHM effects for dietary specialists) and b–f<d–h (SGHM effects for dietary generalists). However the SGHM hypothesis does not offer predictions for the relative magnitude of effects between dietary specialists and generalists. Tests of the main prediction of this hypothesis, that enemy effects will be strongest on herbivores eating low quality plants, have provided mixed empirical support; a meta-analysis [44] suggests that while SGHM effects vary among herbivore and natural enemy guilds, they are strongest for the interactions between predators (vs. parasitoids) and surface-feeding (vs. concealed) herbivores.

### Integrating current hypotheses

There is an emerging perspective that successful theory must explicitly account for the complexities and contingencies in ecological interactions (e.g. [45]). While the PE, EFS and SHGM hypotheses are each integrative to an extent, they fail to consider the simultaneous and possibly interactive (non-additive) effects among host-plant quality, herbivore diet breadth and natural enemies. Such non-additive dynamics would imply that predictions of the PE, EFS, and SGHM hypotheses are each contingent upon the third, discounted factor. Consequently, contingency in the support each hypothesis has received from past empirical tests might be addressed through their consolidation into a single framework.

Accordingly, we present the tri-trophic interactions (TTI) hypothesis. The TTI is based upon the same mechanisms described by existing hypotheses (see above). Novel to the TTI is the integration of these mechanisms in a systematic framework, yielding clear predictions for their combined effects. The TTI makes the central prediction that host-plant quality, herbivore diet breadth and natural enemies interactively determine herbivore performance in ways not explicitly addressed by the PE, EFS and SGHM hypotheses (Fig. 1, Table 1). Although herbivore diet breadth and host plant quality vary continuously, for illustrative purposes it is useful to consider the effects of these factors dichotomously. Specifically, the TTI predicts that dietary specialists (as compared to generalists) escape natural enemies and are competitively dominant due to faster growth rates, and that such differences should be greater on low quality (as compared to high quality) host plants. The mechanistic basis of these predictions can best be understood by considering the TTI from the perspectives of the three component hypotheses it subsumes.

From the first perspective, natural enemies should mediate the predictions of the PE hypothesis. Specifically, the benefits of dietary specialization for herbivore performance are expected to be stronger in the presence than absence of natural enemies due to increased mortality of physiologically inefficient and slow-growing generalists. As depicted in Fig. 1, the TTI hypothesis predicts that e–g≪f–h (PE effects with natural enemies) is greater than a–c<b–d (PE effects without natural enemies).

From the second perspective, host-plant quality should mediate the predictions of the EFS hypothesis. Specifically, the enemy-free space obtained through dietary specialization is expected to be greater on low- than high-quality host plants. Whereas the EFS hypothesis states that specialists avoid natural enemies primarily through crypsis or defense (see above), a narrow diet breadth should also enable natural enemy avoidance by increasing growth rate and thus minimize SGHM-type effects. As depicted in Fig. 1, the TTI hypothesis predicts that c–g≪d–h (EFS effects on low-quality plants) is greater than a–e<b–f (EFS effects on high-quality plants).

Finally, from the third perspective, herbivore diet breadth should mediate the predictions of the SGHM hypothesis. Specifically, plant quality is expected to have a stronger influence over natural enemy-herbivore interactions for dietary generalists than specialist herbivores. As depicted in Fig. 1, the TTI hypothesis predicts that b–d≪f–h (SGHM effects for generalists) is greater and than a–c<e–g (SHGM effects for specialists). Such effects might occur through two complementary mechanisms. Because of their reduced physiological adaptation to their hosts, growth rates of generalists are expected to be relatively sensitive to host-plant quality, and variation in plant defense should strongly mediate the effects of natural enemies. In addition to influencing the window of vulnerability to natural enemies, plant resistance may also differentially affect the crypsis of specialists and generalists. For example, a lower tolerance of generalists for induced plant defense might result in more herbivore movement and dispersed feeding as compared to specialists [46], with such movement in turn increasing predation risk [47].

While the TTI hypothesis predicts that host plant quality and natural enemies mediate the strength of competition between dietary specialists and generalists, it fails to provide an explanation for their coexistence upon shared host plants. Dietary specialists are predicted to outperform generalists under all combinations of natural enemy and host plant quality effects (Fig. 1, Table 1). Although generalists have access to more food resources (e.g. [48]), this in itself cannot explain specialist-generalist coexistence if there are competitively superior specialists on each host plant species. Theory suggests that competitively inferior generalists may persist in fluctuating, non-equilibrium environments [49], [50] (but see [51]), but their ubiquitous co-occurrence with specialists (e.g. [17], [52], [53], [54]) suggests that generalists are competitively superior under certain ecological conditions.

The competitive exclusion of generalists predicted by the TTI hypothesis may explain their relative rarity compared to specialists (e.g. [17], [52], [53], [55]), and also suggests that those generalists that do persist likely employ strategies to provide competitive superiority under some set of ecological conditions. For example, dietary generalists may partly compensate for their relative inability to detoxify plant toxins [28], [56] with more broadly applicable strategies such as suppressing induced responses [57] or deactivating physical defenses [58]. Similarly, a broad diet breadth may afford generalists with strategies for natural enemy avoidance not available to specialists, such as self-medication against parasitoids [59]. Furthermore, while the TTI hypothesis alone does not predict the persistence of generalists, host plant quality and natural enemies may nevertheless interactively determine the conditions under which such generalist strategies may provide for competitive superiority. So while host plant quality and natural enemies may affect specialist and generalist herbivores in the manner described by the TTI hypothesis, theory on specialist-generalist coexistence also predicts that many generalist herbivores will possess one or more strategies that provide for competitive superiority under at least some conditions.

### Empirical test

The integrative nature of the TTI hypothesis presents challenges to empirically testing its predictions, and its evaluation will likely require several complementary approaches. The most rigorous testing will be conducted at the community-level, measuring the effects of natural enemies on assemblages of coexisting herbivores of differing diet breadths, across gradients of intra- or inter-specific variation in host plant quality (e.g. [24], [26]). Alternatively, multiple case studies that each address a small number of specialist and generalist herbivores can be quantitatively synthesized to provide more general conclusions (e.g. [28]).

**Figure 2:**
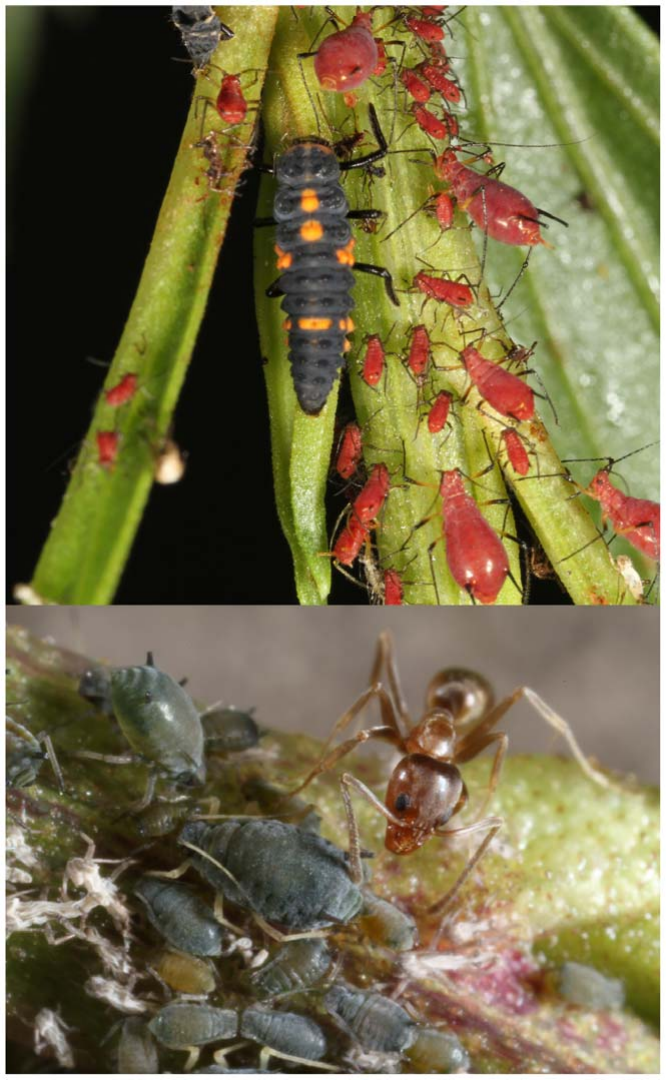
The dietary specialist herbivore *Uroleucon macolai* Blanchard (top) and the generalist *Aphis gossypii* Glover (bottom) feeding upon *Baccharis salicifolia* (Ruiz & Pav.) Pers. Also shown are the predatory ladybird larvae (Coleoptera: Coccinelidae) (top) and the ant *Linepithema humile* (Mayr). Photo credits Kailen Mooney.

Here we provide such a case study and a first test of the TTI hypothesis. Our empirical study investigated two aphid species (Insecta: Hemiptera: Aphididae) (Fig. 2), an extreme dietary specialist (*Uroleucon macolai* Blanchard) and an extreme generalist (*Aphis gossypii* Glover) [60], that locally coexist and feed together on the shared dioecious host plant *Baccharis salicifolia* (Ruiz & Pav.) Pers. With this system, we document the individual and interactive effects of natural enemies and genetically based variation in host-plant quality on the performance of each herbivore. In so doing, we test not only the main predictions of the PE, EFS and SGHM hypotheses, but also the novel predictions of the TTI hypothesis. Our study was also designed to investigate whether these dynamics promote the observed coexistence of these two herbivores on their shared host plant. Specifically, we document the strength and symmetry of competitive interactions between these two aphids, and whether competitive superiority trades off based upon a combination of host plant quality and natural enemies.

## Methods

### Study system

*Baccharis salicifolia* (Asteraceae) is a perennial, woody and dioecious shrub native to the southwestern United States and Northern Mexico. At field sites adjacent to the University of California San Joaquin Marsh Reserve (33.65°N, 117.85°E; Orange County, CA, USA), the two most abundant aboveground herbivores are the aphids *Aphis gossypii* and *Uroleucon macolai* (Mooney, unpublished data). These herbivores co-occur not only at the regional scale, but also within the same *B. salicifolia* stands and occasionally on individual plants and plant stems (Mooney, unpublished data). Flowering, plant growth and the highest herbivore densities occur in March and April. *Aphis gossypii* (Glover) is a generalist herbivore that has an exceptionally wide diet breadth compared to most aphids, feeding on numerous host plant species, including a number of important crop plants [60]. *Uroleucon macolai* has a narrow diet breadth, feeding only on *Baccharis salicifolia* and one other *Baccharis* species [60]. Where *A. gossypii* is frequently tended by ants, *U. macolai* is not (pers. obs). No specific permits were required to work with these plant and insect species, and this work did not involve endangered or protected species.

One well-documented source of variation in plant resistance against herbivores is that frequently found between the sexes within dioecious species [61]. Female plants are predicted to invest more in reproduction than are males [62], [63] and, as they grow more slowly, they are in turn predicted to invest more in defense and to be of lower quality to herbivores [64], [65]. A meta-analysis has found these predictions to generally hold true for chewing herbivores, with females (as compared to males) having slower growth rates, stronger herbivore defenses, fewer herbivores, less herbivore damage, and being of lower quality as determined by herbivore performance [61]. In contrast to chewing herbivores, far less in known of plant sexual dimorphism with respect to sap-feeding herbivores [66] (but see [67]). *Baccharis salicifolia* is primarily defended with mono- and sesquiterpenes [68], which can be important both to plant-aphid and aphid-natural enemy interactions [65], [69], [70].

### Bi-trophic experiment

We cloned seven male and seven female *B. salicifolia* genotypes that originated from a natural population in the University of California San Joaquin Marsh Reserve (33.65°N, 117.85°E) in Orange County, California, USA, and permission of reserve managers was obtained to collect plant material. Clonal copies of parental genotypes originated from 10 cm long stem cuttings of mature plants in June 2009. Cuttings were grown in an outdoor shadehouse in 0.8 L pots (first eight months, through January 2010) and then in 2.0 L pots (last two months, February and March 2010) in a soil mixture of equal parts peat moss, redwood compost, silica sand, and pumice mixed with slow-release fertilizer at a concentration of 0.5 g/L of soil. Aphid populations from multiple field-collected colonies of each species were initiated in February 2010 and maintained on potted *B. salicifolia* in a greenhouse on the UC Irvine campus.

Between 15–17 March 2010 we randomly assigned three *B. salicifolia* plants of each genotype to one of three aphid competition treatments: (1) *U. macolai* alone, (2) *A. gossypii* alone, or (3) a mixed species treatment. Aphids were added to a single growing tip at a density of 20 aphids for single-species treatments and at a density of 10 aphids for each species in mixed-species treatments. Each plant was covered with mesh fabric bags to prevent aphid dispersal. With each aphid treatment being replicated three times per genotype, a total of 126 experimental plants were used (14 plant genotypes×3 aphid treatments×3 replicates = 126). Treatment replicates were stratified across three benches such that benches constituted complete blocks, and the position of plants within a bench was regularly rotated. Plants were watered regularly and maintained at 22–25°C. Aphid populations grew for 35 days at which time final aphid counts were made by one of three people (hereafter “aphid counters”) between 19–23 April.

The competition treatment (single vs. mixed-species) was a substitutive design, and thus tests for differences between intraspecific and interspecific competition [35]. Theory predicts that a species can only persist in a community if intraspecific competition is stronger than interspecific competition [29], [30]. A significant interaction between the effects of competition and either plant genotype or sex on aphid performance indicates that the relative strength of intra- and interspecific competition depends on genetically variable plant traits. If competitive superiority between the aphid species trades off based upon plant genotype or sex, genetic variation in this plant is predicted to promote coexistence.

Aphid performance was measured as per capita daily population growth (*r*, hereafter “population growth rate”), calculated as (ln *N2* - ln *N1*)/(*t2* - *t1*), where *N1* and *N2* are population sizes at time *t1* and *t2*, respectively. Counts of the two aphid species in the mixed species treatment were taken from the same set of plants. Consequently, these data are not independent of each other and separate analyses were performed for each aphid species [35]. For each aphid species, population growth rate was tested for its dependence upon competition (intra- vs. interspecific), either host plant sex or host plant genotype within sex (in separate analyses), and the interaction between competition and host plant sex or host plant genotype. In addition, a separate analysis was conducted to statistically compare the population growth of the two aphid species using data only from single-species treatments. All analyses were conducted using the procedure MIXED in SAS 9.2 [71], with greenhouse bench and aphid counter included as random effects. In analyses of plant genotype effects, plant genotype and genotype×competition interactions were also included as random effects and their significance assessed with log-likelihood ratio tests [72].

### Tri-trophic experiment

On 24 April, following the bi-trophic experiment, 60 of the 126 experimental plants were selected for transfer to a field site at the University of California at Irvine Arboretum (33.66°N, 117.85°E). This site is publically owned by the University of California and no specific permissions were required to work at this location or conduct the described activities. The Arboretum is adjacent to the natural population of *B. salicifolia* from which the experimental plants were originally collected. These 60 plants were randomly selected, with the provision that they equally represented male and female plants of the three aphid competition treatments. While the plants used in this experiment included all of the genotypes from the initial experiment, genotype was not adequately replicated for analysis. Groups of six experimental plants were placed in each of 10 cages of material 70% transparent to light (Lumite Co., Baldwin GA) and 2.5×2.5×2.5 m in size, with each cage including a male and female plant from each of the three aphid competition treatments. Half of the cages were then closed to exclude natural enemies, while the north-facing side of the other cages was left open to allow natural enemy access. Final aphid counts were taken on 15 May, 21 days after the initiation of the experiment.

At the initiation of this field experiment, all plants contained at least some of both aphid species because of aphid dispersal. As a consequence, the distinction between single- and mixed-species treatments was not possible, and the effects of natural enemies were assessed only upon aphids under competition. In addition, plants varied strongly in initial aphid population size. Aphid population growth rate was calculated in the same manner as described above and tested for its dependence upon aphid species, natural enemy exclusion, host plant sex, and their two- and three-way interactions. Because initial population size varied between the two aphid species, aphid performance and the effects of natural enemies might differ due to density dependent effects. Accordingly, the initial population size, and the interaction between initial population size and natural enemy exclusion were also included as fixed effects. In this analysis, initial population size was set to the abundance of both aphid species combined, but preliminary analyses (not shown) demonstrated qualitatively identical results when the initial abundances of each aphid species were included as separate terms. In addition, preliminary analyses (not shown) using a polynomial regression of aphid population growth rate regressed on initial aphid population size showed the higher-order (non-linear) term to be non-significant (P = 0.48). All analyses were conducted using the procedure MIXED in SAS 9.2 [71], with aphid counter, plant identity and cage×natural enemy exclusion interaction included as random effects.

## Results

### Bi-trophic experiment

**Figure 3:**
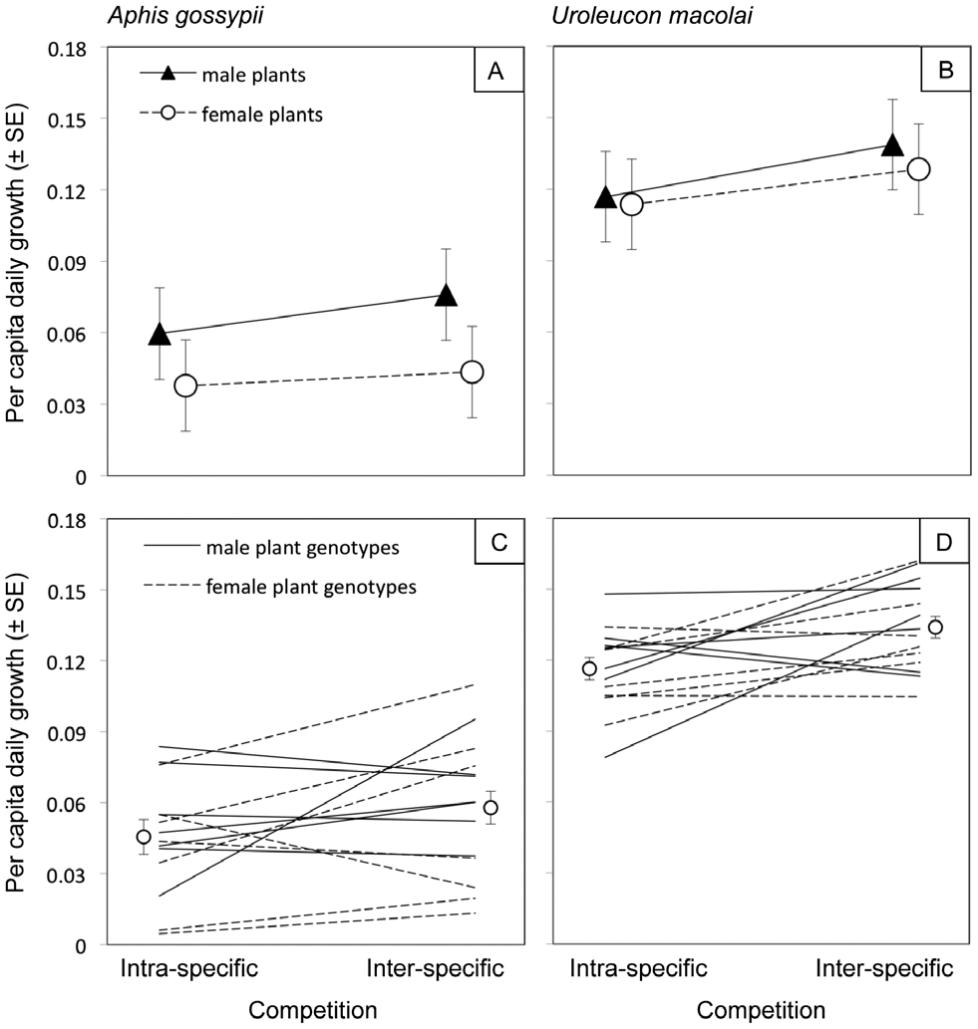
Treatment means from the bi-trophic experiment. Effects of intra- vs. inter-specific competition and genetically based variation in host plant *Baccharis salicifolia* quality on the fecundity of a generalist (*Aphis gossypii*) and a specialist (*Uroleucon macolai*) herbivore. The effects of genetically based variation in host plant quality are tested by comparing herbivore fecundity between male and female plants (panels A, B) and among 14 plant genotypes (panels C, D). For effects of plant sex, means (± 1SE) are shown for each competition treatment. For effects of plant genotype, overall means (± 1SE) are shown for each competition treatment, and means for individual genotypes (error bars omitted for clarity) are indicated with solid (male) or dashed (female) lines. See Table 2 for statistics.

Based on the comparison of single-species treatments, *U. macolai* population growth rate (per capita daily increase) was 240% that of *A. gossypii* (Fig. 3) and after 35 days aphid population size (aphids*plant^−1^) was 550% greater (F_1,61_ = 54.10, P<0.0001) with means (± SEM) for *U. macolai* and *A. gossypii* of 2028±242 and 368±83 respectively. Accordingly, the competitive interaction between the two herbivores was asymmetrical, with the specialist being competitively dominant to the generalist. *Uroleucon macolai* had significantly (16%) higher population growth rate under inter- than intraspecific competition, the outcome predicted to be necessary for persistence in competition with *A. gossypii* (Fig. 3, Table 2). In contrast, *A. gossypii* population growth rate was statistically indistinguishable between treatments of intra- and inter-specific competition (Fig. 3, Table 2). With respect to plant genetic effects, *U. macolai* population growth rate was not influenced by either plant sex or genotype. In contrast, both forms of genetic variation influenced *A. gossypii* performance (Fig. 3, Table 2); population growth rate was 67% higher on male than female plants, and 1040% higher on the highest- as compared to lowest-quality plant genotypes. Despite this substantial variation in host-plant quality for *A. gossypii*, competitive interactions were not mediated by host-plant genotype or sex for either aphid species (Fig. 3, Table 2).

**Table 2:** Test statistics for the bi-trophic experiment.

	*Aphis gossypii*			*Uroleucon macolai*		
Effect	DF	F or χ^2^	P	DF	F or χ^2^	P
*A. Genotype* analysis 1						
Competition (C)	1,13	F = 2.04	0.18	1,13	F = 8.94	0.01
Genotype (sex) (G)^2^	1	χ^2^ = 9.8	0.0017	1	χ^2^ = 1.2	0.27
C×G^2^	1	χ^2^ = 0	1.00	1	χ^2^ = 0.2	0.66
*B. Sex analysis* 1						
Competition (C)	1,72	F = 1.78	0.19	1,73	F = 9.30	0.003
Plant sex (PS)	1,72	F = 10.77	0.0016	1,73	F = 1.24	0.27
C×PS	1,72	F = 0.41	0.52	1,73	F = 0.34	0.56

1 Analysis of both genotype effects (A) and plant sex effects (B) include greenhouse bench and aphid counter as random effects.

2 Tests for the effects of genotype and competition×genotype are made with log-likelihood ratio tests (based upon χ^2^) and genotype is nested within plant sex.

### Tri-trophic experiment

The natural enemies observed in control (open) cages consisted of larval and adult ladybird beetles (Coleoptera, Coccinellidae), parasitic wasps (Hymenoptera, Braconidae) syrphid larvae (Diptera, Syrphidae) and predatory bugs (Hemiptera, Miridae). In addition, ants (*Linepithema humile*), which are otherwise predatory, did not prey upon *A. gossypii* but instead tended them, i.e. collected aphid honeydew [73] in both open and closed cages.

At the initiation of the experiment, the mean population size (aphids*plant^−1^) for *U. macolai* was similar between male and female plants (female: 1587±289; male: 1825±349; overall: 1706±225) because plant sex did not influence its performance in the bi-trophic experiment. In contrast, the superior performance of *A. gossypii* on male plants meant the initial mean populations differed more substantially between male and female plants (female: 124±32; male: 237±73; overall: 180±40). After 21 days, population sizes fell in all treatments for both *U. macolai* (male, no predator: 584±126; male, predator: 55±41; female, no predator: 408±82; female predator 25±17) and *A. gossypii* (male, no predator: 136±64; male, predator: 7±4; female, no predator: 54±22; female, predator 12±6).

**Figure 4:**
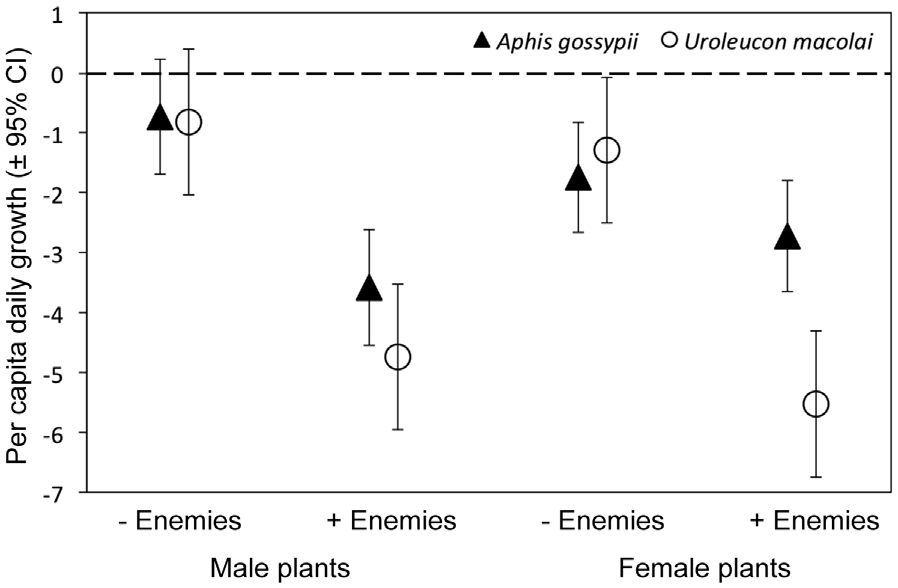
Treatment means from the tri-trophic experiment. Effects of natural enemies and *Baccharis salicifolia* sex on the fecundity of a generalist, ant-tended herbivore (*Aphis gossypii*) and a specialist, untended herbivore (*Uroleucon macolai*). Least square means (±95% CI) for per capita daily growth are shown for each treatment controlling for initial aphid population size. See Table 3 for statistics.

Population growth rate was influenced by initial aphid density and by natural enemy exclusion, but these two effects operated independently, i.e. predation was not density-dependent (Table 3). Controlling for variation in aphid density, there was a three-way interaction between the effects of natural enemies, aphid species and plant sex on population growth rate (Table 3). To explore the basis of this three-way interaction, we inspected the least square mean ±95% CI (controlling for initial aphid density) of population growth rate for each aphid species, in treatments with and without natural enemies, on male and female plants (Fig. 4). This three-way interaction can be viewed from three complementary perspectives.

**Table 3:** Test statistics for the tri-trophic experiment.

Effect^1^	DF	F	P
Natural enemies (NE)	1,8	18.53	0.0026
Plant sex (PS)	1,56	0.63	0.43
Aphid species (AS)	1,56	7.68	0.0076
NE×PS	1,56	2.19	0.15
NE×AS	1,56	10.29	0.0022
PS×AS	1,56	1.31	0.26
NE×PS×AS	1,56	4.47	0.039
Initial density	1,56	4.21	0.0448
Initial density×NE	1,56	0.05	0.82

1 Aphid counter, plant identity and cage×natural enemy exclusion interaction are included as random effects.

First, the two-way interaction between plant sex and aphid species (i.e. plant quality×herbivore diet breadth, the PE hypothesis) was mediated by the presence of natural enemies; in the absence of natural enemies, plant sex had equivalent effects on the two herbivores (94% greater rate of population decline on female vs. male plants), while in the presence of natural enemies the effect of plant sex differed, with *A. gossypii* performing better on female than male plants (31% greater rate of population decline on male vs. female plants), and *U. macolai* performing better on male than female plants (17% greater rate of population decline on female vs. male plants).

Second, the two-way interaction between natural enemies and aphid species (i.e. natural enemies×herbivore diet breadth, the EFS hypothesis) was mediated by plant sex (i.e. host plant quality); for male plants, natural enemies had equivalent effects on both aphid species (430% greater rate of population decline with natural enemies vs. without), while on female plants natural enemies had strong negative effects on *U. macolai* (330% greater rate of decline with natural enemies vs. without) but weaker effects on *A. gossypii* (56% greater rate of decline in with natural enemies vs. without).

And third, the two-way interaction between natural enemies and host plant sex (i.e. natural enemies×plant quality, the SGHM hypothesis) was mediated by aphid species (i.e. herbivore diet breadth); for the dietary specialist *U. macolai*, the effects of natural enemies was equally strong on both female and male plants (385% greater rate of population decline with natural enemies vs. without), while for the generalist *A. gossypii* the effect of natural enemies was stronger on male (386% greater rate of population decline with natural enemies vs. without) than female plants (56% greater rate of population decline with natural enemies vs. without).

## Discussion

The PE, EFS and SGHM hypotheses are each predicated upon separate, two-way interactions between host plant quality, herbivore diet breadth and natural enemies (Fig. 1, Table 1), and they have been highly influential due to their integrative nature. Yet individually and collectively, these hypotheses do not consider the simultaneous effects of all three factors. While a definitive test of the TTI hypothesis requires studying multiple specialist and generalist herbivores, here we provide a preliminary assessment comparing individual specialist and generalist aphid herbivores on a shared host plant. Our results suggest that the dynamics predicted by these past hypotheses are non-independent, and thus the need for integrative theory. The TTI predicts that dietary specialists (as compared to generalists) escape natural enemies and are competitively dominant due to faster growth rates, and that such differences should be greater on low quality (as compared to high quality) host plants. These predictions were supported in part, and the observed herbivore population dynamics were better explained by the TTI hypothesis than by its three subsidiary hypotheses. Our results also speak to the factors promoting the coexistence of dietary specialist and generalist herbivores, suggesting that a trade-off in competitive superiority is based upon the interactive combination of both host plant quality and natural enemies. We discuss the explanatory power of this new hypothesis, and the novel insights it provides.

### Tests of component hypotheses

The central prediction of the PE hypothesis of dietary specialists having superior performance than generalists on shared host plants (Fig. 1, a>b, c>d, e>f, and g>h) was supported experimentally (Fig. 3). In the bi-trophic experiment, the specialist *U. macolai* maintained a reproductive rate more than twice that of the generalist *A. gossypii*. This same comparison can also be made in the predator-free cages of the tri-trophic experiment. While the difference in performance was much less pronounced here, with *A. gossypii* populations having a 17% lower reproductive rate than *U. macolai*, these experimental conditions were far more variable than those of the bi-trophic experiment, and the latter provides a more rigorous test of the PE hypothesis. This finding is consistent with the notion that dietary specialization enhances efficiency of food utilization [1], [24].

Consistent with PE hypothesis predictions that plant quality should have stronger effects on generalists than specialists (Fig. 1: a–c<b–d, PE effects without natural enemies; e–f<g–h, PE effects with natural enemies), the generalist *A. gossypii*, but not the specialist *U. macolai*, varied in performance based upon both plant sex and genotypic variation within plant sex (Fig. 3). Male plants were of higher quality than females, consistent with the general patterns observed from past studies [61]. These findings parallel past work showing that generalists are more sensitive to variation in plant defense than specialists (e.g. [26], [28]). The potential for plant genotypic differences to structure arthropod communities is well recognized [74], including competitive interactions among herbivores (reviewed by [35], [38]) and herbivore interactions with mutualists [75] and natural enemies [75], [76]. Our findings suggest that plant genetic effects on herbivore performance are likely to be stronger for generalist than specialist herbivores.

Yet our assessment of natural enemy effects (Fig. 4) produced results at odds with predictions; the effects of natural enemies were equivalent for the two herbivores on high-quality (male) plants, but relatively weak for the generalist *A. gossypii* on low-quality (female) plants (Fig. 4). So while the EFS hypothesis predicts stronger natural enemy effects on generalists than specialists (Fig. 1: a–e<b–f, EFS effects on high-quality plants; c–g<d–h, EFS effects on low-quality plants), there were either no differences in natural enemy effects between the herbivores (on high-quality male plants) or stronger natural enemy effects on the specialist (on low-quality female plants). Similarly, the SGHM hypothesis predicts stronger natural enemy effects on low-quality host plants (Fig. 1: a–e<c–g, SGHM effects for specialists; b–f<d–h, SGHM effects for generalists), yet there was either no difference in natural enemy effects based on plant quality (for the specialist *U. macolai*) or stronger natural enemy effects on high-quality male plants (for the generalist *A. gossypii*).

### Test of the tri-trophic interactions hypothesis

As predicted by the TTI, there was a three-way interaction among the effects of host-plant quality, herbivore diet breadth and natural enemies. That the generalist *A. gossypii* but not the specialist *U. macolai* varied in performance between plant sexes had tri-trophic consequences, as plant sex mediated natural enemy effects for the former but not the latter (Fig. 4). This result demonstrates the inadequacy of considering only the pairwise effects of these factors, and underscores the need for integrative theory.

Yet our experiment produced an overall pattern of mixed support for the more specific TTI predictions. The TTI hypothesis predicts that PE effects should be greater with natural enemies (Fig. 1., e–g≪f–h) than without natural enemies (Fig. 1, a–c<b–d); consistent with this prediction, the performance of the two herbivores differed more in the presence than absence of natural enemies, and on low-quality female plants than high-quality male plants; contrary with this prediction, however, the performance of the generalist *A. gossypii* was better, not worse, than the specialist *U. macolai* on the low-quality female plants with natural enemies. The TTI hypothesis also predicts that EFS effects should be greater on low-quality plants (Fig. 1, c–g≪d–h) than on high-quality plants (Fig. 1, a–e<b–f); consistent with this prediction, natural enemies influenced the relative performance of the two herbivores more on low-quality female than high-quality male plants, and this effect was due to the generalist *A. gossypii* (but not the specialist *U. macolai*) being sensitive to host-plant quality; contrary with this prediction, natural enemy effects were weaker – not stronger – for the generalist *A. gossypii* on low-quality female plants. Finally, the TTI hypothesis predicts that SGHM effects should be greater for generalists (Fig. 1, b–d≪f–h) than for specialists (Fig. 1, a–c<e–g); consistent with this prediction, plant quality mediated the effects of natural enemies for the generalist *A. gossypii* but not the specialist *U. macolai*, and it was on low-quality female plants where natural enemy effects differed between the two herbivores; contrary with this prediction, natural enemy effects were weaker – not stronger – for the generalist *A. gossypii* on low-quality female plants.

In each case, departure from TTI predictions was due to the generalist *A. gossypii* performing better than expected on low-quality female plants in the presence of natural enemies. We reason that the unexpectedly high performance of *A. gossypii* on female plants in a tri-trophic context was due to an additional element of this particular tri-trophic system, namely the mutualistic interaction between this aphid and ants [73]. *Aphis gossypii*, but not *U. macolai*, engages in mutualistic interactions with ants. Ant tending can improve aphid performance by providing protection from natural enemies [73] and removal of competing herbivores [35], but the strength of such benefits declines with increasing aphid density (e.g. [77]). Therefore, the superior performance and higher density of *A. gossypii* on male plants in a bi-trophic context may have resulted in weaker ant protection from natural enemies. And because ant attendance carries physiological costs for aphids [78], the net effect of ants on high-quality male plants may have been neutral, or even negative. For instance, Mooney and Agrawal [75] documented that ant effects on aphids were positive on low-quality milkweed genotypes, but negative on high-quality genotypes (but see [79]). Consequently, a strong benefit of ants to *A. gossypii* on low-quality female plants, coupled with costs of ants on high-quality male plants and in the absence of natural enemies, may underlie these unanticipated results.

Our test of the TTI hypothesis thus identifies a possible strategy of the generalist *A. gossypii* that underlies its ability to coexist with the specialist *U. macolai*. The bi-trophic experiment showed that *U. macolai* was competitively superior to, and likely to exclude *A. gossypii*, and this was true across all male and female host plant genotypes. Without mutualist ants, we speculate that these two herbivores would respond to host plant quality and natural enemies as predicted by the TTI hypothesis, with *A. gossypii* being competitively excluded and driven locally extinct. Importantly, even under ant protection *Aphis gossypii* only demonstrated superior performance on low-quality plants and with natural enemies present, demonstrating that coexistence depends upon trade-offs that extend across multiple niche axes [35], [80]. These findings are similar to those from milkweed, where *Aphis asclepiadis* escapes competitive exclusion by *Aphis nerii* due to an interactive combination of superior performance on select host plant genotypes [35], [75], tending by mutualist ants [75], and differences in aphid phenologies [81]. More generally, our results demonstrate the utility of the TTI in explaining herbivore coexistence as compared to existing hypotheses that are narrower in scope.

### Conclusions

Well-tested theory has demonstrated that host plant quality, natural enemies and diet breadth influence herbivore performance. Our preliminary test of the TTI hypothesis demonstrates that these factors interactively determine herbivore performance in ways not explicitly addressed by the PE, EFS and SGHM hypotheses, and that the integration of these hypotheses is relevant to competitive interactions and coexistence among herbivores. Future studies should additionally consider the potentially different roles played by predators and parasitoids, which may respond differently to variation in herbivore quality between high and low-quality host plants [44], [82]. Similarly, the consequences of continuous variation in both host plant quality and herbivore diet breadth should be addressed.

Several of the predictions of the TTI hypothesis were unmet, with the generalist herbivore demonstrating superior performance under a subset of conditions circumscribed by both host plant quality and natural enemies. Importantly, this departure from predictions may be due to the very mechanism allowing for the observed coexistence between this specialist and generalist herbivores. Because the TTI predicts the competitive exclusion of generalist herbivores, results from case studies of generalist and specialist herbivores that do, in fact, coexist are likely to individually produce results that deviate from expectations. Accordingly, a strong test of the TTI hypothesis is only likely to come from the synthesis of results across many systems, where the signal of the predicted pattern may be detected through the noise generated by the requirements for coexistence. Such synthesis might come from factorial studies comparing multiple pairs of specialist and generalist herbivores, community-level studies in which the interactive effects of natural enemies and host plant quality are assessed for herbivore assemblages of varying diet breadths (e.g. [26]), as well as meta-analytic approaches that synthesize the results of past empirical studies (e.g. [28]). Through this process, these splintered hypotheses in plant-herbivore and herbivore-enemy interactions can be unified into a broader, more inclusive theory of tri-trophic interactions.
